# The Influence of Genotype and Seasonality on the Sow Colostrum Quality and Immunoglobulin G Content

**DOI:** 10.3390/ani15121802

**Published:** 2025-06-18

**Authors:** Kristina Gvozdanović, Vice Čuljak, Vladimir Margeta, Ivona Djurkin Kušec, Boris Antunović, Dalida Galović, Goran Kušec

**Affiliations:** 1 Department for Animal Science and Biotechnology, Faculty of Agrobiotechnical Sciences Osijek, 31000 Osijek, Croatia; vmargeta@fazos.hr (V.M.); idurkin@fazos.hr (I.D.K.); boris.antunovic@fazos.hr (B.A.); dalidag@fazos.hr (D.G.); gkusec@fazos.hr (G.K.); 2 State Inspectorate, Šubičeva 29, 10000 Zagreb, Croatia; vice.culjak@gmail.com

**Keywords:** pig, colostrum, piglet, interaction, proteins

## Abstract

The aim of this study was to investigate the influence of genotype (GT) and seasonality (NP) on the quality parameters of sow colostrum and evaluate the efficiency of the radial immunodiffusion (RID) analysis and the Brix refractometer in determining the IgG concentration. This study was conducted on 240 sows that originated from two genotypes, namely GT1 (TOPIGS, n = 120) and GT2 (Pig Improvement Company, n = 120), during the three farrowing periods: the winter farrowing period (WNP, n = 80), the summer farrowing period (SMP, n = 80) and the spring farrowing period (SSP, n = 80). The significant interaction effect was observed for protein (*p* < 0.0001), lactose (*p* < 0.05) and non-fat solids (SNT) (*p* < 0.001). At the same time, the interaction effect influenced the IgG concentration measured with the Brix refractometer (*p* < 0.0001) and RID (*p* < 0.0001). Pearson’s correlation coefficient showed that Brix percentage was positively correlated with RID results (r = 0.52, *p* < 0.0001), while the Bland–Altman plots indicated a mean bias of −1.93. Partial eta-squared analysis (η^2^) showed that the genotype explained the largest proportion of variance in fat content (η^2^ = 0.136) and IgG concentration (η^2^ = 0.164), while interaction effects were largest for protein (η^2^ = 0.072). The results of this study show that genotype and seasonality influence sow colostrum quality, which indicates the importance of genotype−seasonality interactions in breeding programs for optimizing the colostrum quality and piglet survival.

## Simple Summary

Colostrum is the first milk that piglets consume after farrowing. It is rich in various nutrients, the most important of which are immunoglobulins, which are responsible for the development of immunity. The composition of colostrum is influenced by the genotype and changes depending on the season. The results of this study showed that the genotype and season significantly influence the colostrum fat and protein contents, while the lactose content varied only by genotype. The highest fat and protein concentrations were observed during the winter period, indicating seasonal adaptation. We also found that the Brix refractometer is a reliable and on-farm method for determining the IgG concentration in the colostrum of sows. Selecting the specific genotypes based on seasonal performance could enhance colostrum quality and also improve piglet health and survival rates. This could benefit pig producers by improving piglet survival rates, animal welfare and potentially economic performance.

## 1. Introduction

Colostrum is the secretion of the mammary glands and is the piglets’ first food. It is rich in immunoglobulins, peptides and growth factors, which are crucial for the survival of newborn pigs [1]. The specificity of colostrum is its high concentration of proteins and its low content of fat and lactose [2]. The production of colostrum and later milk influences the growth, survival and development of piglets in the later stages of production. Considering that piglet mortality can range from 11% to 24% [3], it is important to analyze the composition of colostrum and its possible role in reducing mortality. The role of colostrum in the early life stage of piglets is to promote the passive immunity of piglets [4], provide the energy for thermoregulation [5] and participate in the development of the intestinal system [2]. Previous studies have reported that litter size, the number of parities, nutrition, genetics and the management system have an influence on colostrum composition [6,7]. Also, some studies have shown that variation in seasonal temperature can have an effect on the colostrum composition [8,9]. Nuntapaitoon et al. [8] reported that heat stress to which sows are exposed during the summer months leads to a decrease in concentration of immunoglobulins in colostrum, while during the colder period the levels of immunoglobulins increased. Amatucci et al. [10] and Picone et al. [11] reported that the different composition of colostrum also depends on the genotype of the sow. Their results showed that the colostrum from Duroc sows have higher concentrations of immunoglobulin G (IgG) and immunoglobulin A (IgA) compared to sows originated form Landrace and Large White breeds.

The composition of immunoglobulin G (IgG) in colostrum varies between pig breeds and sows, which has an impact on piglet survival. IgG is the predominant protein component in colostrum, followed by immunoglobulin G (IgA) and immunoglobulin M (IgM), the concentrations of which are much higher in colostrum than in mature milk. According to Quesnel et al. [12] and Hurley [6], the IgG content in colostrum is around 64 mg/mL and is often higher in colostrum than in sow plasma [13]. The concentration of IgG and IgA decreases exponentially in the first 24 h after birth. It has been found that dead piglets have a lower serum IgG concentration [14], suggesting that colostrum intake also has a major impact on piglet survival. To reduce piglet mortality, it is important to measure the IgG concentration in colostrum. Also, the quality of the colostrum is assessed on the basis of the IgG concentration in its composition. It is considered to be of high quality if the colostrum contains ≥50 g IgG/L [15]. Various techniques have been developed to measure the IgG concentration. Direct methods measure the actual IgG concentration and include the enzyme-linked immunosorbent assay (ELISA) [16], the radial immunodiffusion (RID) test [17], chromatography [18] and electrophoresis [19]. In contrast, indirect methods such as refractometry [20] and turbidimetric immunoassays [21] are based on a rapid indirect determination of the IgG concentration. Radial immunodiffusion (RID) is the most commonly used technique for the determination of IgG concentration. RID is a classical method whose performance is based on the determination of the antigen or antibody concentration in an unknown sample [15]. This method has been used to determine the concentration of immunoglobulins in colostrum in various animal species such as sows [20,22,23], cows [16,24], horses [25,26] and goats [27,28]. Although this method is expensive, error-prone and time-consuming, it is considered the gold standard for determining the IgG concentration in colostrum. The use of the RID method is not suitable for on-farm use as it takes 18 to 24 h for the results to be available. Therefore, alternative methods for determining the IgG concentration in colostrum were investigated [22].

The estimation of the IgG concentration in colostrum using the Brix refractometer is based on analyzing the total solids in non-sugar-containing liquids, while optical density correlates with the IgG content in colostrum [29]. It is a quick and inexpensive method, and for these reasons it is intended as a practical method for assessing colostrum quality on the farm. Several authors have compared the effectiveness of different methods for determining immunoglobulin concentration in the colostrum of domestic animals [20,22,30,31]. Balzani et al. [20] tested the Brix refractometer as an on-farm method for assessing the immunoglobulin G concentration in sow colostrum. The authors concluded that the positive correlation between Brix and RID values shows that the use of a Brix refractometer is a practical farm tool for determining the immunoglobulin concentration. Gamsjäger et al. [32] compared the Brix refractometer with the RID test to determine the IgG concentration in the colostrum of cattle. Their results showed a positive correlation between these two methods and confirmed that Brix refractometry is a promising on-farm tool for the determination of IgG concentration in colostrum.

While the importance of colostrum for piglets from the aspect of their survival and immunity has been previously study [13,33,34], the influence of genotype–season interactions on colostrum quality parameters remains underexplored. Considering the importance of the quality and nutrient profile of colostrum on piglet survival, it is important to understand these specific relations between genotypes and challenging climate changes. Thus, the aim of this study was to investigate the influence of seasonality and genotype on the chemical composition of colostrum in the first 24 h after birth and compare the efficiency of the RID method and the Brix refractometer in determining the IgG concentration.

## 2. Materials and Methods

### 2.1. Animals

The experimental protocol was approved by the Bioethics Committee of the Faculty of Agrobiotechnical Sciences Osijek (2158–94–02–25–08), and all procedures were performed in accordance with the Croatian Animal Welfare Act and other legal acts regulating animal husbandry and welfare.

The study was conducted on 240 sows, which were divided into two groups according to their genotype, GT1 and GT2. The sows in the first group (GT1) belonged to the Topigs Norsvin TN70 (Den Bosch, The Netherlands) (Landrace × Large White, TP, n = 120), and the sows in the second group (GT2) belonged to the Camborough Pig Improvement Company (Hendersonville, TN, USA) (Large White × Landrace, PIC, n = 120). Colostrum samples were taken in the winter farrowing period from December to February, in the spring farrowing period from March to May and in the summer farrowing period from June to August. Within each genotype, colostrum samples were collected during the three farrowing periods: the winter farrowing period (WNP, n = 40), the summer farrowing period (SMP, n = 40) and the spring farrowing period (SSP, n = 40). The sows were kept under the same housing conditions in groups of 20 sows. During the study period, the sows were fed a commercial gestation diet containing 13.00 MJ/ME and 12.00 g/kg crude protein (2.45 kg feed/day), and twice daily they had free access to water. In addition to natural light, artificial light was used for 8 to 16 h per day, depending on the season, with the exception of farrowing day when it was 24 h per day. The temperature in the barn was between 18° C and 22° C, and the humidity was between 70% and 80%. One week before farrowing, the animals were moved to a farrowing pen and kept in individual pens (2.0 × 2.5 m) on a plastic slatted floor. The sows were not fed on farrowing day.

### 2.2. Colostrum Sampling and Statistical Analysis

Colostrum samples were collected within 0–3 h after birth. To ensure adequate hygiene, each teat was washed with warm water and dried before sampling. Colostrum was collected evenly from the first anterior pair of teats in 10 mL conical vials. After sampling, the colostrum was immediately frozen at −20 °C until further analysis. To proceed with the RID, the colostrum samples were melted in the laboratory and placed in radial immunodiffusion plates for a 24 h incubation period. The result of the analysis was a precipitin ring whose diameter was measured and compared with the standard curve obtained by measuring the diameter of the precipitin circles formed by the reaction between anti-immunoglobulins and colostrum samples with a known amount of immunoglobulins. Prior to analysis, the Brix refractometers (HANNA HI 96801 refractometer, HANNA Instruments, Woonsocket, RI, USA) were calibrated with distilled water. For further analysis, 0.5 mL of fresh and mixed colostrum was placed on the prism of the BRIX refractometer, and the Brix percentage (%) was recorded. The total solids and immunoglobulin G (IgG) levels in the colostrum were measured twice to determine the repeatability of the results. The primary analysis for the fat, protein, lactose and non-fat solids (SNT) of the colostrum was determined using the Milkoscan FT 120 (FOSS, Hillerod, Denmark).

### 2.3. Statistical Analysis

Statistical analyses were performed using the package dplyr [35] for data manipulation and the packages ggplot2 [36], FactoMineR [37], factoextra [38] and car [39] for visualization, and the Bland–Altman method was used as a visual tool in the R environment (Version 4.0.2) [40]. All numerical data are presented as mean values with the standard error of the mean (SEM). The dataset was checked, and redundant values were excluded from further analysis. The normal distribution of the data and the homogeneity of variance were tested using the Shapiro–Wilk test and the Levene test. A two-way ANOVA with the genotype and season as fixed factors was applied in the analysis of effects of seasonality and genotype on colostrum chemical composition and IgG concentration. When ANOVA showed statistically significant differences, pairwise comparisons were performed using Tukey’s honestly significant difference (HSD) post hoc test to identify specific differences between genotype and season combinations within a single parameter. Statistical significance was determined at a 95% confidence interval, and the difference was considered significant at the *p* < 0.05 level. Partial eta-squared (η^2^) values were calculated and visualized to estimate the proportion of variance explained by genotype and season, as well as their interaction, with thresholds for small (η^2^ = 0.01) and moderate (η^2^ = 0.06) effects.

## 3. Results

The genotype had a significant effect on fat (*p* < 0.0001), protein (*p* < 0.001) and lactose (*p* < 0.0001), while the season affected only the fat (*p* < 0.0001) and protein (*p* < 0.05) content (Table 1). However, no significant seasonal influence on the lactose and solids-not-fat (SNT) content was observed (*p* > 0.05). The fat content was highest in both genotypes in the WNP (GT1: 5.62 and GT2: 4.96). In contrast, the lowest fat content was found in the summer farrowing period (SMP) (GT1: 5.05 and GT2: 3.87) and was statistically different from the SSP and WNP. The protein content showed significant differences that were related to both genotype (*p* < 0.001) and season (*p* < 0.05). GT1 shows a significant seasonal response with increasing values from the SSP (14.72) to the WNP (16.20). Both the GT1 and GT2 genotypes exhibit intermediate levels of protein content during the SMP (15.50 and 15.53, respectively). The highest protein levels were observed in the SSP for GT2 and the WNP for GT1 (16.55 and 16.20, respectively). The lactose content ranged from 2.56 to 2.67 for GT1 and from 2.27 to 2.43 for GT2. Results on SNT levels show that this value does not vary significantly between genotypes and seasons (*p* > 0.05). The highest value for GT1 was observed in the SMP (20.52), followed by the SSP (18.77) and WNP (18.52). For GT2, the highest SNT values were observed in the WNP (20.18) and the lowest in the SMP (18.38).

**Figure 1:**
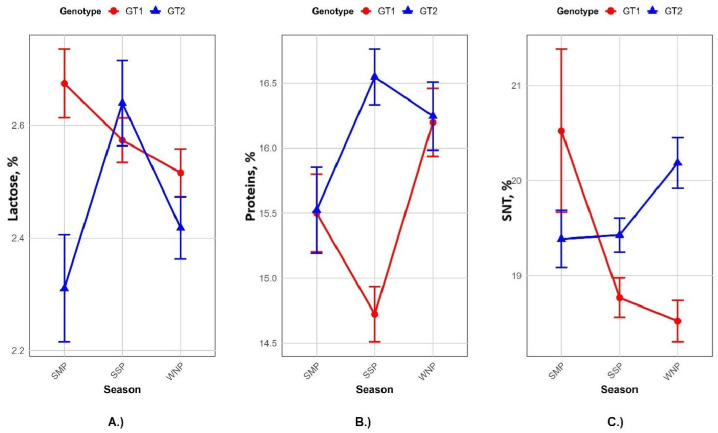
Season (SMP, SSP and WNP) by genotype (GT1 and GT2) interaction for lactose (**A**), protein (**B**) and SNT (**C**) content; spring farrowing period = SSP; summer farrowing period = SMP; winter farrowing period = WNP; GT1 = TOPIGS; and GT2 = Pig Improvement Company.

Figure 1 shows the interaction effect between seasons and genotypes for the analyzed parameters. Each graph shows the interaction between a specific season (WNP, SMP or SSP) and the genotype GT1 or GT2. The significant interaction was observed for protein (*p* < 0.0001), lactose (*p* < 0.03) and SNT (*p* < 0.001). Both genotypes have similar protein levels in the SMP (15.50 for GT1 and 15.53 for GT2) but differences occur during the SSP, in which GT1 exhibited the lowest values while GT2 had the highest protein content. As for the lactose content, no statistically significant differences (*p* > 0.05) were found in relation to the season effect, although there were differences between genotypes (*p* < 0.0001). The genotype and season have no influence on SNT content, but their interaction has an impact on SNT levels.

**Figure 2:**
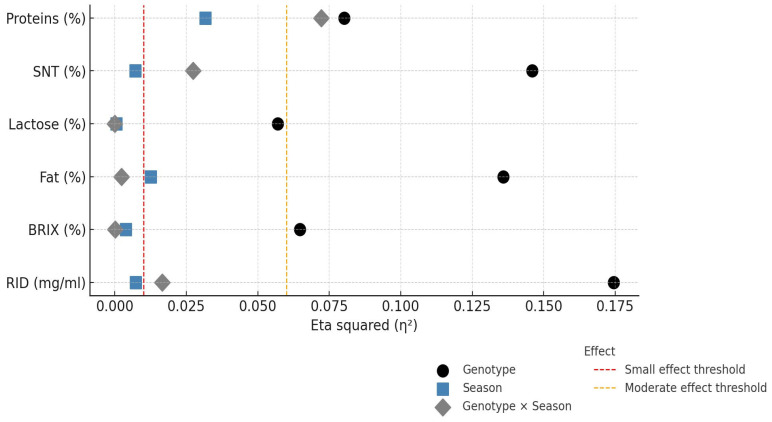
Effect sizes (η^2^) for genotype, season and their interaction for fat, lactose, protein and solids-not-fat content (SNT); refractometer Brix values; and IgG concentrations. Horizontal dashed lines indicate thresholds for small (η^2^ = 0.01) and moderate (η^2^ = 0.06) effects. Traits are labeled with the corresponding measurement units.

**Figure 3:**
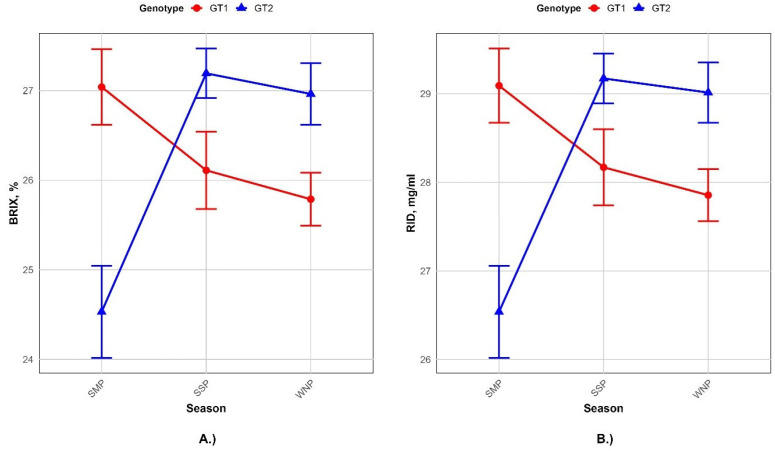
Season (SMP, SSP and WNP) by genotype (GT1 and GT2) interaction for BRIX (**A**) and the RID refractometer regarding (**B**) the value of IgG concentrations; SSP = spring farrowing period; SMP = summer farrowing period; WNP = winter farrowing period; TP = TOPIGS sows; and PIC = Pig Improvement Company sows.

The analysis of effect sizes (η^2^) (Figure 2) showed that the genotype explained most of the variance in the fat content (η^2^ = 0.136) and IgG concentration (RID method; η^2^ = 0.164), indicating a strong genetic influence on these parameters. The season showed moderate effects on the protein content (η^2^ = 0.030) and a minimal effect on RID values (η^2^ = 0.007). Interaction effects were small for most of the analyzed traits, except for protein content. Interaction charts presented on Figure 1 and Figure 3 show the relative performance of two genotypes for the analyzed colostrum quality traits across seasons.

BRIX values for GT1 differed scientifically across the seasons (*p* = 0.05), but no effect of genotype (*p* > 0.05) was found between GT1 and GT2. The IgG BRIX value in GT1 during the SMP (27.04) was significantly different from the SSP (26.11) and WNP (25.84) (Table 2). In contrast, the IgG BRIX value in GT2 was highest for the SSP (27.24) and differed from the IgG BRIX values in the SMP (24.43) which had the lowest immunoglobulin content. The obtained results showed that the genotype has no significant effect (*p* > 0.05) on IgG values measured using the RID method, while the seasonal influence was marginal (*p* = 0.05). The highest IgG RID values in GT1 were recorded for the SMP (29.09) and are significantly higher than those for the SSP (28.17) and WNP (27.85). These high values during the summer farrowing period may reflect an adaptive response to environmental demands. In contrast, the SMP in GT2 had the lowest RID value (26.53) and was significantly different from the WNP (29.04) and SSP (29.17). Also, the IgG content for GT1 in the SMP indicates its ability to produce immune-rich colostrum compared with GT2 during the same period.

The significant interaction between the genotype and season was observed for both the concentration of IgG BRIX (*p* < 0.0001) as well as for the IgG concentration measured by the RID method (*p* < 0.0001) (Figure 3). Although the interaction effect for the BRIX value is significant, the values for GT1 differ between seasons, similar to the IgG BRIX values in the GT2 genotype. This distinct peak of IgG concentration in the SSP and WNP observed in both genotypes reflects the ability of GT2 to accumulate soluble solids during mid-season conditions. A similar trend was observed for the IgG concentrations measured using the RID refractometer. The GT2 genotype exhibited increased values during the SSP and WNP, while the decreasing trend for all the seasons was observed for GT1. The obtained results showed that IgG values measured using both the RID value and BRIX are significantly influenced by the season. However, information on the influence of the interaction between the pig genotype and the season on colostrum composition and IgG concentration is relatively scarce and remains largely undetermined.

**Figure 4:**
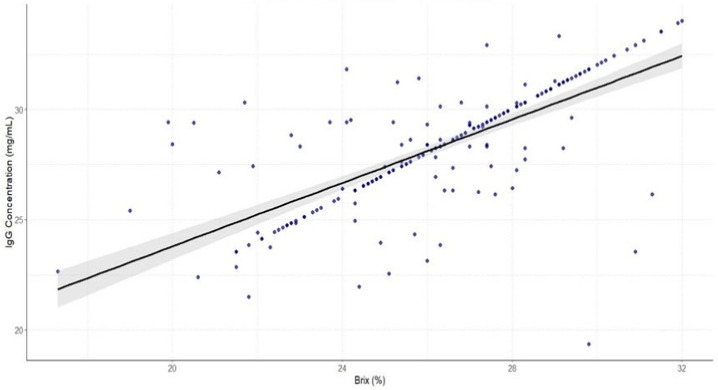
Regression plot between the concentration of immunoglobulin G (IgG) by radial immunodiffusion (RID) (mg/mL) and the refractometer Brix value (%) of the analyzed samples. The gray area represents the confidence interval of the mean value.

The results shown on Figure 4 show a positive linear relationship between IgG concentrations measured by two different methods (r = 0.52, *p* < 0.0001), suggesting that the Brix refractometer can potentially be used as a non-invasive, rapid predictor of IgG levels in sow colostrum.

**Figure 5:**
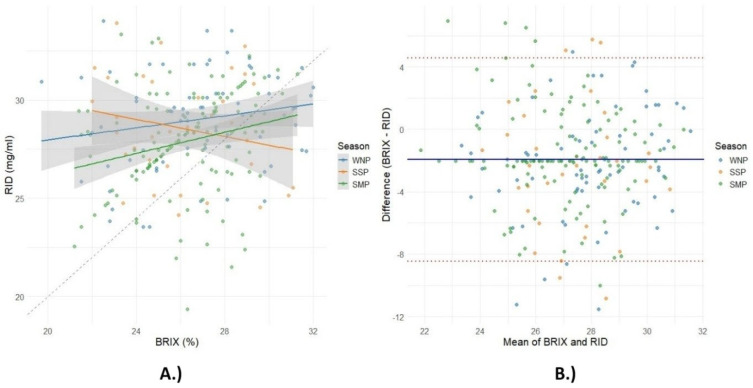
Regression (**A**) and Bland–Altman plots (**B**) of the concentration of immunoglobulin G (IgG) by radial immunodiffusion (RID) (mg/mL) and the refractometer Brix value (%) regarding the season (spring farrowing period = SSP; summer farrowing period = SMP; and winter farrowing period = WNP).

The seasonal effect (*p* < 0.05) was significant for both RID and BRIX IgG levels (Table 2), and the linear regression model showed a positive relationship between RID and BRIX IgG during the different time periods (Figure 4). The regression model showed that R2 was highest for the WNP (R2 = 0.32), indicating a moderate positive correlation between BRIX and RID, followed by the SSP (R2 = 0.28) and SMP, which showed a weak and less consistent relationship (R2 = 0.15). Figure 5B shows the results of the Bland–Altman analysis, which assesses the agreement between the Brix refractometer and the RID method by plotting the differences against the average of the two methods.

## 4. Discussion

Our results showed that the genotype has an effect on the protein, fat and lactose content, whilst the season influenced only fat and proteins (Table 1). Several studies have shown that different environmental factors occurring during different seasons can alter metabolic processes, leading to differences in the composition of colostrum, especially in terms of the fat and protein content [1,11]. The obtained study showed that the fat content was the highest during the winter period, and in the summer period it was the lowest. Similar results for a lower fat content during the summer season were found by Viridis et al. [41]. This decrease in fat content during the warm season could be due to the higher ambient temperatures. High environmental temperatures during summer have a negative effect on nutrient absorption and fatty acid synthesis. This is particularly pronounced in lactating sows. Qu and Ajuwon [42] and Qu et al. [43] found that high temperatures lead to the realizing of free fatty acids from adipose tissue to prevent the loss of body fat reserves. In addition, energy is utilized to maintain body temperature rather than lactation, which affects the energy substrates available for colostrum synthesis [44]. Dado-Senn et al. [45] stated that microstructural changes that occur in mammary tissue during high temperatures alter milk and colostrum synthesis. These changes disrupt proteins involved in maintaining the tight junctions of the epithelium and impair nutrient transport processes crucial for milk production [46]. As a result, the quality and functional composition of this fat is altered, leading to lower fatty acid profiles and an altered colostrum composition [47].

The genotype is one of the factors that can influence the composition of sow colostrum, as already reported in several studies [31,48,49,50]. Hasan et al. [31] and Hasan et al. [51] reported lower values for fat (4.5 and 4.6) and higher values for lactose (5.4 and 4.1) and protein (15.9 and 17.0) in TOPIGS sows compared to our results. In agreement with the present study, Declerck et al. [49] reported significant effects of the different genotypes on the colostral fat content. The authors also conclude that the observed effect of genotype on fat content in sow colostrum is an important selection strategy to improve the colostrum composition. In addition, Picone et al. [11], Trevisi et al. [52] and Nuntapaitoon et al. [7] reported higher fat and protein content in the colostrum of Large White and Landrace sows compared to Duroc sows. The observed differences are primarily attributed to genetic predispositions and breed-specific metabolic pathways. The genetic regulation of nutrient transporters and lipid metabolism is enriched in the mammary tissue of Large White and Landrace sows, which leads to better mammary gland function during lactation compared to Duroc sows [53]. Better proliferation of mammary epithelial cells and improved lipid synthesis in the mammary glands lead to the production of high-quality colostrum in Large White and Landrace sows [42]. The better metabolic adaptability of Large White and Landrace sows affects the processing and storage of nutrients and thus has a direct influence on the resulting nutrient composition of the milk [9].

The genotype and season exhibited an interaction effect (Figure 1) on the protein content in colostrum, which is likely due to a seasonal adaptation where increased protein levels provide structural or metabolic benefits under colder conditions. Viridis et al. [41] reported similar results for protein levels and found that both fat and protein levels were higher in the colder season than in the warmer season. The authors argued that this could be a result of environmental influences on the hormonal changes in the sows and increased protein synthesis in the colostrum. In contrast to our results, Declerck et al. [49] found no influence of genotype on the lactose and protein content. The seasonal effect was not significant for the lactose content (*p* > 0.05), but our study found that the genotype has an influence on lactose in the colostrum. Previous studies were consistent with ours, and differences in lactose content among different seasons were not observed [54,55]. In contrast, Virdis et al. [41] reported differences in lactose content throughout the seasons and attributed that to the adaptive responses to environmental conditions.

The results of the partial eta-squared (η^2^) analysis (Figure 2) show the greatest influence of the genotype on the fat content (η^2^ = 0.136) and the IgG concentration determined using the RID method (η^2^ = 0.164). The results are consistent with those in Table 1. Similarly, an interaction effect was observed for protein content (η^2^ = 0.072), which manifested itself in a seasonally specific genotype (SSP) response. These results confirm that the effect of genotype on protein content is not linear across seasons but is modulated by environmental conditions. For lactose, although a statistically significant genotype effect was confirmed (*p* < 0.0001) (Table 1), the proportion of variance explained by seasonal and interaction effects was small (η^2^ < 0.01). The variability in SNT values confirms the genotype–season interaction and emphasizes the need for an integrated interpretation of the effects. The season had a moderate effect, particularly on protein content, which is consistent with the observed seasonal variation. The results obtained on lactose content, both in terms of the genotype and seasonal effects, are consistent with the results by Picone et al. [11]. In their study, the authors found significant breed-related differences in lactose content (*p* > 0.0001), although they did not observe a significant effect of the season on the lactose content (*p* = 0.136). Hasan et al. [31] also reported that both the genotype and season significantly influence the fat, protein and lactose content in the colostrum.

According to Farmer and Quesnel [48], Quesnel [1], Declerck et al. [49] and Amatucci et al. [10], the concentrations of immunoglobulins in the colostrum vary by genotype. Large White and Landrace sows have a genetic predisposition for the synthesis and secretion of higher levels of immunoglobulins into the colostrum [43]. In contrast, Duroc sows do not have this genetic predisposition for the regulation of nutrient transporters and lipid metabolism within the mammary tissue. Contrary to that, Amatucci et al. [10] reported genetic predispositions in Duroc sows for producing colostrum with higher immunoglobulin concentrations compared to the Large White and Landrace breeds. Also, Picone et al. [11] and Trevisi et al. [52] reported significant differences in IgG concentrations in the colostrum of Large White, Landrace and Duroc sows, while Hasan et al. [31] found differences between four different sow breeding lines (DanAvl, Topigs, Duroc and Norwegian Landrace). The observed breed differences in the immunoglobulin content in the colostrum can influence the proliferation and activity of mammary epithelial cells, as well as the efficiency of immunoglobulin transfer from the sow’s bloodstream into the colostrum.

Some studies also showed the influence of season on the immunoglobulin content in sow colostrum [31,48,56,57]. Juthamanee et al. [57] and Nuntapaitoon et al. [8] analyzed IgG concentrations in high-producing sows reared in a warm environment. The authors found that IgG concentrations during the warm season ranged from 50.2 mg/mL to 59.5 mg/mL [57] and 60.8 mg/mL to 79.1 mg/mL [8], respectively. Farmer and Quesnel [49] found that IgG increases in spring and decreases in summer and autumn, which is consistent with our results. In contrast, Bernabucci et al. [55] reported a lower IgG concentration during the summer period in sows under heat stress. Higher environmental temperatures in summer can reduce feed intake and change the hormonal status of the sow, particularly the cortisol and prolactin levels [58]. This has a negative effect on the immune response and the transfer of immunoglobulins to the mammary gland. In addition, increased body temperature and stress in late gestation can impair the normal immune response and reduce the amount of IgG available for transfer in the colostrum [59].

Repeatability coefficients were calculated for IgG measured using the BRIX and RID methods (Table 2). The results showed low coefficients for both BRIX (0.0189)- and RID (0.0151)-measured IgG concentration, suggesting that the seasonal effect is stronger than the genetic effect, as indicated by the obtained *p*-value (0.05 for the season and 0.07 for the genotype). These high values during the summer farrowing period may reflect an adaptive response to environmental demands. Also, the IgG content for GT1 in the SMP indicates its ability to produce immune-rich colostrum compared with GT2 during the same period. This shows that the IgG concentration in sows’ colostrum is more stable in different genotypes, while the performance fluctuates more depending on the seasonal conditions.

Regression analysis showed a positive linear relationship between IgG concentrations measured by two different methods (Figure 4). The determination of IgG concentration in colostrum is crucial for the assessment of colostrum quality and important in terms of the impact on pig production due to piglet survival [60]. Several authors have used the Brix refractometer as an effective tool for estimating the immunoglobulin G (IgG) concentration in the colostrum and compared its accuracy with traditional methods such as radial immunodiffusion (RID) [20,61], the enzyme-linked immunosorbent assay (ELISA) [22,30,62], the colostrometer [63] and infrared (IR) spectroscopy [64]. The results by Balzani et al. [20] showed that the percentages of BRIX values were positively correlated with the IgG results of the radial immunodiffusion method, with an R2 of 0.31, which is lower than in our study. The reason for the differences could be the calibration of the refractometers used in the studies or differences in colostrum sampling and treatment. Hasan et al. [62] and Stojić et al. [65] also show a positive correlation between BRIX and RID in determining IgG concentrations, suggesting that the Brix value can effectively determine colostrum IgG levels.

These seasonal differences in the regression parameters highlight the different correlation and predictability between BRIX and RID (Figure 5A), with stronger correlations observed in the WNP than in the SMP. Bland–Altman analysis (Figure 5B) is useful to identify systematic bias and the range of random error [66]. Combined linear regression analysis and Bland–Altman analysis showed a significant relationship between the two methods for determining the IgG concentration in the colostrum, which was strongest for the WNP. The mean bias was calculated to be −1.93, indicating that the difference between the BRIX measurements and the RID values was 1.93 units, indicating lower BRIX values. The upper limit was 4.58, and the lower limit was −8.45, which corresponds to a 95% deviation between the two methods. The presence of a non-zero mean deviation indicates that there is a systematic discrepancy between the two methods [67]. The seasonal differences were also observed from the distribution around the mean difference line, with the WNP showing larger dispersion.

## 5. Conclusions

The results of this study show that the genotype and season and their interaction influenced the quality parameters of colostrum. Both the genotype and season had an effect on the fat and protein content, while lactose was only influenced by the genotype. A significant interaction effect was observed for protein, lactose and SNT. GT1 had a higher fat content across all seasons, while GT2 was superior for protein content and immunoglobulin content as measured by the RID method.

The positive correlation between the BRIX and RID measurements showed that the Brix refractometer can be used as a practical method for the on-farm assessment of IgG concentration in pig colostrum. Overall, the results showed that the GT1 genotype has better results for fat content, especially during the summer period, while GT2 is superior when measuring protein and immunoglobulin IgG content using the RID method in spring and summer. This is supported by the effect size analysis (η^2^), which highlighted the genotype as the dominant factor for colostrum traits and also confirms the relevance of genotype–season interactions, especially for protein composition. It can be concluded that the interaction between the genotype and season plays an important role in sow colostrum quality, but it also emphasizes the need for research on specific breeding strategies and refining measurement techniques to improve colostrum quality and reduce piglet mortality under difficult environmental conditions. Future research should include longitudinal monitoring of colostrum and the analysis of immunoglobulins A and M and the effects of targeted supplements during late pregnancy.

## Figures and Tables

**Table 1:** Mean values and standard error of the mean of colostrum for different genotypes and seasons of sampling.

Trait	Fat, %	SEM	Proteins, %	SEM	Lactose, %	SEM	SNT, %	SEM
	GT1							
SSP	5.49 ^a^	0.13	14.72 ^bc^	0.19	2.57	0.04	18.77	0.19
SMP	5.05 ^b^	0.21	15.50 ^b^	0.31	2.67	0.05	20.52	0.86
WNP	5.62 ^a^	0.12	16.20 ^a^	0.25	2.56	0.04	18.52	0.20
	GT2							
SSP	4.64 ^a^	0.14	16.55 ^a^	0.22	2.43	0.05	19.43	0.18
SMP	3.87 ^b^	0.15	15.53 ^b^	0.32	2.27	0.09	19.38	0.29
WNP	4.96 ^a^	0.19	16.25 ^a^	0.26	2.42	0.06	20.18	0.27
Genotype	0.0001		0.001		0.0001		0.24	
Season	0.0001		0.05		0.88		0.11	

SNT = solids-not-fat, %; SSP = spring farrowing period; SMP = summer farrowing period; WNP = winter farrowing period; GT1 = TOPIGS; and GT2 = Pig Improvement Company. ^a,b,c^ Significant differences (*p* < 0.05) between groups are denoted by different lowercase letters.

**Table 2:** Mean values and standard error of the mean IgG concentration measured using the radial immunodiffusion (RID) method and the Brix refractometer (BRIX) for different genotypes and seasons of sampling.

Trait	RID, mg/mL	SEM	BRIX, %	SEM
GT1				
SSP	28.17 ^b^	0.45	26.11 ^b^	0.43
SMP	29.09 ^a^	0.40	27.04 ^a^	0.42
WNP	27.85 ^b^	0.27	25.84 ^b^	0.29
GT2				
SSP	29.17 ^a^	0.27	27.24 ^a^	0.28
SMP	26.53 ^b^	0.51	24.43 ^b^	0.22
WNP	29.04 ^a^	0.99	26.96 ^a^	0.34
Genotype	0.70		0.81	
Season	0.05		0.05	
Repeatability	0.0151		0.0189	

SSP = spring farrowing period; SMP = summer farrowing period; WNP = winter farrowing period; GT1 = TOPIGS; GT2 = Pig Improvement Company; and RID = radial immunodiffusion. ^a,b^ Significant differences (*p* < 0.05) between groups are denoted by different lowercase letters

## Data Availability

None of the data were deposited in an official repository. The data can be provided by the corresponding author upon reasonable request.

## References

[1] Quesnel H. (2011). Colostrum production by sows: Variability of colostrum yield and immunoglobulin G concentrations. Animal.

[2] Inoue R., Tsukahara T. (2021). Composition and physiological functions of the porcine colostrum. Anim. Sci. J..

[3] Oliviero C. (2013). Management to improve neonate piglet survival. J. Reprod. Infertil..

[4] Charneca R., Nunes J.T., Freitas A., Le Dividich J. (2021). Effect of litter birth weight standardization before first suckling on colostrum intake, passive immunization, pre-weaning survival, and growth of the piglets. Animal.

[5] Villanueva-García D., Mota-Rojas D., Martínez-Burnes J., Olmos-Hernández A., Mora-Medina P., Salmerón C., González-Lozano M. (2020). Hypothermia in newly born piglets: Mechanisms of thermoregulation and pathophysiology of death. J. Anim. Behav. Biometeorol..

[6] Hurley W.L., Farmer C. (2015). Composition of sow colostrum and milk. The Gestating and Lactating Sow.

[7] Nuntapaitoon M., Juthamanee P., Theil P.K., Tummaruk P. (2020). Impact of sow parity on yield and composition of colostrum and milk in Danish Landrace× Yorkshire crossbred sows. Prev. Vet. Med..

[8] Nuntapaitoon M., Suwimonteerabutr J., Am-In N., Tienthai P., Chuesiri P., Kedkovid R., Tummaruk P. (2019). Impact of parity and housing conditions on concentration of immunoglobulin G in sow colostrum. Trop. Anim. Health Prod..

[9] Amavizca-Nazar A., Montalvo-Corral M., González-Rios H., Pinelli-Saavedra A. (2019). Hot environment on reproductive performance, immunoglobulins, vitamin E, and vitamin A status in sows and their progeny under commercial husbandry. J. Anim. Sci. Technol..

[10] Amatucci L., Luise D., Correa F., Bosi P., Trevisi P. (2022). Importance of breed, parity and sow colostrum components on litter performance and health. Animals.

[11] Picone G., Zappaterra M., Luise D., Trimigno A., Capozzi F., Motta V., Trevisi P. (2018). Metabolomics characterization of colostrum in three sow breeds and its influences on piglets’ survival and litter growth rates. J. Anim. Sci. Biotechnol..

[12] Quesnel H., Farmer C., Theil P.K., Farmer C. (2015). Colostrum and milk production. The Gestating and Lactating Sow.

[13] Maciag S.S., Bellaver F.V., Bombassaro G., Haach V., Morés M.A.Z., Baron L.F., Bastos A.P. (2022). On the influence of the source of porcine colostrum in the development of early immune ontogeny in piglets. Sci. Rep..

[14] Vallet J.L., Miles J.R., Rempel L.A. (2013). A simple novel measure of passive transfer of maternal immunoglobulin is predictive of preweaning mortality in piglets. Vet. J..

[15] Westhoff T.A., Behling-Kelly E.L., Mann S. (2024). Comparison of radial immunodiffusion, turbidimetric immunoassay, and Brix refractometry for determining bovine colostrum quality. JDS Commun..

[16] Dunn A., Duffy C., Gordon A., Morrison S., Argűello A., Welsh M., Earley B. (2018). Comparison of single radial immunodiffusion and ELISA for the quantification of immunoglobulin G in bovine colostrum, milk and calf sera. J. Appl. Anim. Res..

[17] Niero G., Thomas S.A., Mouratidou K., Visentin G., De Marchi M., Penasa M., Cassandro M. (2023). Lactoferrin concentration in bovine milk: Validation of radial immunodiffusion technique, sources of variation, and association to udder health status. Ital. J. Anim. Sci..

[18] Wang L., Zhou L., Ma N., Su Q., Wan Y., Zhang Y., Qian W. (2022). Real-time monitoring of immunoglobulin G levels in milk using an ordered porous layer interferometric optical sensor. Talanta.

[19] Stastna M., Šlais K. (2023). Preparative separation of immunoglobulins from bovine colostrum by continuous divergent-flow electrophoresis. J. Sep. Sci..

[20] Balzani A., Cordell H.J., Edwards S.A. (2016). Evaluation of an on-farm method to assess colostrum IgG content in sows. Animal.

[21] Sievert M., Schuler G., Büttner K., Wehrend A. (2022). Comparison of different methods to determine the absorption of colostral IgG in newborn foals. J. Equine Vet. Sci..

[22] Souza A.P., Bombassaro G.E., Fonseca F.N., Lopes L.D.S., Maciag S.S., Volpato F.B., Bastos A.P. (2021). A comparative evaluation of methods for estimating the colostrum quality in sows. Arq. Bras. Med. Vet. Zootec..

[23] Schoos A., De Spiegelaere W., Cools A., Pardon B., Van Audenhove E., Bernaerdt E., Maes D. (2021). Evaluation of the agreement between Brix refractometry and serum immunoglobulin concentration in neonatal piglets. Animal.

[24] Breuer R.M., Wiley C., Dohlman T., Smith J.S., McKeen L., Kreuder A.J. (2023). Comparison of turbidometric immunoassay and brix refractometry to radial immunodiffusion for assessment of colostral immunoglobulin concentration in beef cattle. J. Vet. Intern. Med..

[25] Turini L., Bonelli F., Nocera I., Meucci V., Conte G., Sgorbini M. (2021). Evaluation of different methods to estimate the transfer of immunity in donkey foals fed with colostrum of good IgG quality: A preliminary study. Animals.

[26] de Lima T.C., de Sobral G.G., de França Queiroz A.E.S., Chinelate G.C.B., Porto T.S., Oliveira J.T.C., Carneiro G.F. (2024). Characterization of lyophilized equine colostrum. J. Equine Vet. Sci..

[27] Shao X., Cheng M., Zhang X., Wang C., Jiang H. (2021). Analysis on the effect of the various factors on immunoglobulin G in goat colostrum. Proceedings of the 2020 International Conference on Environmental Engineering and Energy Science and Technology (IAECST 2020).

[28] Zamuner F., Cameron A.W.N., Carpenter E.K., Arcos-Gómez G., Leury B.J., DiGiacomo K. (2024). Postponing first colostrum collection: Impact on immunoglobulin G in goat colostrum. Animal.

[29] McCue P.M. (2014). Colostrum banking. Equine Reproductive Procedures.

[30] Quigley J.D., Lago A., Chapman C., Erickson P., Polo J. (2013). Evaluation of the Brix refractometer to estimate immunoglobulin G concentration in bovine colostrum. J. Dairy Sci..

[31] Hasan S., Orro T., Valros A., Junnikkala S., Peltoniemi O., Oliviero C. (2019). Factors affecting sow colostrum yield and composition, and their impact on piglet growth and health. Livest. Sci..

[32] Gamsjäger L., Elsohaby I., Pearson J.M., Levy M., Pajor E.A., Haines D.M., Windeyer M.C. (2020). Assessment of Brix refractometry to estimate immunoglobulin G concentration in beef cow colostrum. J. Vet. Intern. Med..

[33] Oliviero C., Junnikkala S., Peltoniemi O. (2019). The challenge of large litters on the immune system of the sow and the piglets. Reprod. Domest. Anim..

[34] Farmer C., Edwards S.A. (2022). Improving the performance of neonatal piglets. Animal.

[35] Yarberry W., Yarberry W. (2021). CRAN Recipes: DPLYR, Stringr, Lubridate, and RegEx in R.

[36] Wickham H., Chang W., Wickham M.H. (2016). Package ‘ggplot2’. Create Elegant Data Visualisations Using the Grammar of Graphics. Version 2. https://ggplot2.tidyverse.org/reference/ggplot2-package.html.

[37] Husson F., Josse J., Le S., Mazet J., Husson M.F. (2016). Package ‘Factominer’. Version 2.11. https://cran.r-project.org/web/packages/FactoMineR/index.html.

[38] Kassambara A., Mundt F. Package ‘Factoextra’. Extract and Visualize the Results of Multivariate Data Analyses. R Package Version. https://rpkgs.datanovia.com/factoextra/.

[39] Fox J., Weisberg S., Adler D., Bates D., Baud-Bovy G., Ellison S., Heiberger R. (2012). Package ‘Car’. https://cran.r-project.org/web/packages/car/index.html.

[40] R Development Core Team (2020). *R. A Language and Environment for Statistical Computing*, Version 4.0.2. Computer Software : Vienna, Austria. https://www.R-project.org.

[41] Virdis S., Luise D., Correa F., Laghi L., Arrigoni N., Amarie R.E., Trevisi P. (2024). Productive and metabolomic consequences of arginine supplementation in sows during different gestation periods in two different seasons. J. Anim. Sci. Biotechnol..

[42] Qu H., Ajuwon K.M. (2018). Metabolomics of heat stress response in pig adipose tissue reveals alteration of phospholipid and fatty acid composition during heat stress. J. Anim. Sci..

[43] Qu H., Yan H., Lu H., Donkin S.S., Ajuwon K.M. (2016). Heat stress in pigs is accompanied by adipose tissue–specific responses that favor increased triglyceride storage. J. Anim. Sci..

[44] Xin W.U., Li Z.Y., Jia A.F., Su H.G., Hu C.H., Zhang M.H., Feng J.H. (2016). Effects of high ambient temperature on lipid metabolism in finishing pigs. J. Integr. Agric..

[45] Dado-Senn B., Skibiel A.L., Fabris T.F., Dahl G.E., Laporta J. (2019). Dry period heat stress induces microstructural changes in the lactating mammary gland. PLoS ONE.

[46] Tao S., Orellana R.M., Weng X., Marins T.N., Dahl G.E., Bernard J.K. (2018). Symposium review: The influences of heat stress on bovine mammary gland function. J. Dairy Sci..

[47] Ma X., Jiang Z., Zheng C., Hu Y., Wang L. (2015). Nutritional regulation for meat quality and nutrient metabolism of pigs exposed to high temperature environment. J. Nutr. Sci..

[48] Farmer C., Quesnel H. (2009). Nutritional, hormonal, and environmental effects on colostrum in sows. J. Anim. Sci..

[49] Declerck I., Dewulf J., Piepers S., Decaluwé R., Maes D. (2015). Sow and litter factors influencing colostrum yield and nutritional composition. J. Anim. Sci..

[50] Quesnel H., Resmond R., Merlot E., Père M.C., Gondret F., Louveau I. (2023). Physiological traits of newborn piglets associated with colostrum intake, neonatal survival and preweaning growth. Animal.

[51] Hasan S., Saha S., Junnikkala S., Orro T., Peltoniemi O., Oliviero C. (2019). Late gestation diet supplementation of resin acid-enriched composition increases sow colostrum immunoglobulin G content, piglet colostrum intake and improve sow gut microbiota. Animal.

[52] Trevisi P., Luise D., Won S., Salcedo J., Bertocchi M., Barile D., Bosi P. (2020). Variations in porcine colostrum oligosaccharide composition between breeds and in association with sow maternal performance. J. Anim. Sci. Biotechnol..

[53] Chen F., Zhang S., Deng Z., Zhou Q., Cheng L., Kim S.W., Guan W. (2018). Regulation of amino acid transporters in the mammary gland from late pregnancy to peak lactation in the sow. J. Anim. Sci. Biotechnol..

[54] Renaudeau D., Noblet J. (2001). Effects of exposure to high ambient temperature and dietary protein level on sow milk production and performance of piglets. J. Anim. Sci..

[55] Bernabucci U., Basiricò L., Morera P. (2013). Impact of hot environment on colostrum and milk composition. Cell. Mol. Biol..

[56] Inoue T., Kitano K., Inoue K. (1980). Possible factors influencing the immunoglobulin G concentration in swine colostrum. Am. J. Vet. Res..

[57] Juthamanee P., Suwimonteerabutr J., Tummaruk P. (2024). The influence of parity, body condition, litter size and carbetocin administration on colostrum production and immunoglobulin levels in highly productive sows within a tropical environment. Trop. Anim. Health Prod..

[58] Patra M.K., De U.K., Kent Y., Rungsung S., Krishnaswamy N., Deka B.C. (2021). Influence of seasonal variation on post-farrowing dysgalactia syndrome (PFDS) and serum biochemistry profiles in the periparturient sow. Trop. Anim. Health Prod..

[59] Szabó C., Ortega A.D.S.V., Lugata J.K., Czeglédi L., Csernus B., Gulyás G., Horváth M. (2025). Factors Affecting the Ig Content of Sow’s Colostrum: A Systematic Review and Meta-Analysis. Agriculture.

[60] Quesnel H., Farmer C., Devillers N. (2012). Colostrum intake: Influence on piglet performance and factors of variation. Livest. Sci..

[61] Papatsiros V., Argyris G., Papakonstantinou G., Meletis E., Tsekouras N., Kantas D., Kostoulas P. (2022). Evaluation of an on-farm method to assess colostrum IgG content in hyperprolific sows. Anim. Reprod. Sci..

[62] Hasan D., Ford H.R., Bionaz M. (2025). Impact of maternal diet on the antioxidant status, immune function, and whole-blood selenium levels of lamb offspring. Ital. J. Anim. Sci..

[63] Röder M., Borchardt S., Heuwieser W., Rauch E., Sargent R., Sutter F. (2023). Evaluation of laboratory and on-farm tests to estimate colostrum quality for dairy cows. J. Anim. Sci..

[64] Elsohaby I., McClure J.T., Dow N., Keefe G.P. (2018). Effect of heat-treatment on accuracy of infrared spectroscopy and digital and optical brix refractometers for measuring immunoglobulin G concentration in bovine colostrum. J. Vet. Intern. Med..

[65] Stojić M., Fratrić N., Kovačić M., Ilić V., Gvozdić D., Savić O., Đoković R. (2017). Brix refractometry of colostrum from primiparous dairy cows and new-born calf blood serum in the evaluation of failure of passive transfer. Acta Vet..

[66] Acar T.O. (2024). Comparing Measurement Reliability Estimation Techniques: Correlation Coefficient vs. Bland–Altman Plot. Meas-Interdiscip. Res. Perspect..

[67] Mansournia M.A., Waters R., Nazemipour M., Bland M., Altman D.G. (2021). Bland-Altman methods for comparing methods of measurement and response to criticisms. Glob. Epidemiol..

